# A profile of prognostic and molecular factors in European and Māori breast cancer patients

**DOI:** 10.1186/1471-2407-10-543

**Published:** 2010-10-10

**Authors:** Gabi U Dachs, Maiko Kano, Ekaterina Volkova, Helen R Morrin, Valerie CL Davey, Gavin C Harris, Michelle Cheale, Christopher Frampton, Margaret J Currie, J Elisabeth Wells, Bridget A Robinson

**Affiliations:** 1 Angiogenesis and Cancer Research Group, University of Otago, Christchurch, New Zealand; 2 Cancer Society Tissue Bank, University of Otago, Christchurch, New Zealand; 3 Christchurch Breast Cancer Patient Register, Christchurch Hospital, Christchurch, New Zealand; 4 Anatomical Pathology, Christchurch Hospital, Christchurch, New Zealand; 5 Department of Medicine, University of Otago, Christchurch, New Zealand; 6 Public Health and General Practice, University of Otago, Christchurch, New Zealand; 7 Oncology Services, Christchurch Hospital, Christchurch, New Zealand

## Abstract

**Background:**

New Zealand Māori have a poorer outcome from breast cancer than non-Māori, yet prognostic data are sparse. The objective of this study was to quantify levels of prognostic factors in a cohort of self-declared Māori and European breast cancer patients from Christchurch, New Zealand.

**Methods and Results:**

Clinicopathological and survival data from 337 consecutive breast cancer patients (27 Māori, 310 European) were evaluated. Fewer tumours were high grade in Māori women than European women (p = 0.027). No significant ethnic differences were detected for node status, tumour type, tumour size, human epidermal growth factor receptor, oestrogen and progesterone receptor (ER/PR) status, or survival.

In addition, tumour and serum samples from a sub-cohort of 14 Māori matched to 14 NZ European patients were analyzed by immunohistochemistry and enzyme linked immunosorbent assay for molecular prognostic factors. Significant correlations were detected between increased grade and increased levels of hypoxia inducible factor-1 (HIF-1α), glucose transporter-1 (GLUT-1), microvessel density (MVD) and cytokeratins CK5/6 (p < 0.05). High nodal status correlated with reduced carbonic anhydrase IX (CA-IX). Negative ER/PR status correlated with increased GLUT-1, CA-IX and MVD. Within the molecular factors, increased HIF-1α correlated with raised GLUT-1, MVD and CK5/6, and CK5/6 with GLUT-1 and MVD (p < 0.05). The small number of patients in this sub-cohort limited discrimination of ethnic differences.

**Conclusions:**

In this Christchurch cohort of breast cancer patients, Māori women were no more likely than European women to have pathological or molecular factors predictive of poor prognosis. These data contrast with data from the North Island NZ, and suggest potential regional differences.

## Background

Breast cancer remains the most common malignancy in New Zealand women, with over 2400 women diagnosed each year [1]. The current trend of earlier detection, due to regular mammography, and increased use of adjuvant chemotherapy have improved breast cancer survival, yet almost half of women with localised breast cancer develop metastases [2] and over 600 women each year in New Zealand die from their disease [1]. Standard prognostic indicators for breast cancer, as recognized by the National Cancer Institute in 1990, include lymph node status, tumour size, nuclear grade, hormone receptor status, tumour type, and human epidermal growth factor receptor (Her2) status [3].

The lack of oxygen (hypoxia) in breast tumours has been proposed as an additional prognostic and predictive marker [4,5]. Tissue hypoxia leads to an adaptive response regulated via hypoxia inducible factor-1 (consisting of HIF-1α and HIF-1β) [6], and raised HIF-1α levels have been associated with reduced survival, chemotherapy failure, relapse and risk of metastases in breast cancer [5,7,8].

Tissue banking to collect cancer tissue for research was initiated in 1996 by clinicians and scientists at Christchurch Hospital and University of Otago, Christchurch [9]. The Cancer Society Tissue Bank (CSTB) now holds tissue samples from over 4500 consented cancer patients, including samples from over 1000 women with breast cancer. The collection of ethnicity data is relatively recent, having been introduced in 2003 [9]. Although the incidence rate of breast cancer is reportedly similar across the two main ethnic groups in New Zealand, age-standardised (World Health Organisation) mortality rate of breast cancer is significantly higher in Māori women (36.2/100 000) than in non-Māori/non-Pacific women (mostly European, 24.5/100 000) [10,11]. In addition, 5-year relative survival ratios are reportedly lower in Māori (74%) than in non-Māori/non-Pacific women (83% [12]).

The causes underlying the ethnic disparity in cancer outcomes in New Zealand are unknown. High mortality from all types of cancer in Māori have been attributed to deprivation [13] and socioeconomic status [14], low health care utilisation [12], and presence of risk factors such as smoking and obesity [15]. Several national studies have demonstrated that Māori women had a higher likelihood of disease spread at diagnosis [12-14,16]. It has been suggested that the difference in stage at diagnosis, localised disease vs. regional or distant spread, is one of the major factors contributing to the ethnic disparity in mortality and survival [12,17]. However, a report to the Ministry of Health has shown that adjusting for stage at diagnosis accounted for only one third of the survival difference [11]. A recent study of Auckland women with breast cancer suggested that Māori women presented with a more aggressive disease (high grade, large size tumours with increased lymph node involvement) [18].

Internationally, there is increasing evidence that molecular factors may be contributing to discrepancies in cancer survival between different ethnicities [19]. For example, African-American women tend to get breast cancer at a much younger age and exhibit different tumour characteristics, such as a negative hormonal receptor status, which is frequently associated with positive axillary lymph node status even in small tumours, and high nuclear grade [20-22]. In addition, Her2 status in African American breast cancer patients did not predict outcome, unlike in European patients [22].

These reports have led us to investigate the clinicopathological and molecular features of breast cancer patients with self-identified Māori and European ethnicity within the Christchurch population.

## Methods

### Human ethics

Ethical approval for this study was granted by the Upper South B Regional Ethics Committee, New Zealand (Ethic approval numbers URB/05/08/102 and URB/06/10/078). The use of tissue and serum samples donated to CSTB was approved by the CSTB Board. All participants have given written informed consent.

### Breast cancer patients

As ethnicity data collection was first implemented on the 12^th ^of May 2003 [9], data from all female breast cancer patients who have gifted tissue to the CSTB between 12^th ^of May 2003 and 31^st ^of December 2007 was investigated. Follow-up was recorded until 14^th ^of September 2009. The tissue banking consent form offers the declaration of ethnicity according to the New Zealand Census (2001/2006) format, and as such includes individuals with multiple ethnic affiliations. For this study, a prioritised ethnic classification system was used where individuals were classified as Māori if Māori was self-reported as one of the ethnic groups in any ethnicity field. Of the 443 female breast cancer patients on the CSTB database for this time period, 67 did not supply ethnicity data, and 20 were of other ethnicities representing groups too small to be analysed separately, and were thus excluded. Patients with ductal carcinoma in situ (DCIS) but no invasive carcinoma (n = 12), and patients with evidence of prior cancer (breast or other, according to their medical notes, n = 7), were also excluded. Of the remainder, 310 women were self-declared European (including NZ European and Other European) and 27 women were self-declared Māori (including 17 sole Māori, i.e. those who identified with no other ethnicity). Ethnicity of the cohort is shown in Table 1.

**Table 1:** Ethnicity data of breast cancer patients.

Self-declared Ethnicity	**No**.	Ethnic classification		
Māori	16	Māori		
Māori/Indian or NZ European/Māori or NZ European/Māori/Kiwi	11	Māori		
NZ European	290		European	
Other, American Caucasian or American Irish or British or Dutch or English or European or German or Irish or New Zealander or Scottish or Russian or NZ European/British or Dutch	20		European	
Other, Filipino or Thai or Australian or Malaysian Chinese or Chinese	15			excluded
Samoan or NZ European/Samoan	5			excluded
No ethnicity stated	67			excluded

**Total**	**424**	**27**	**310**	**(87)**

Ethnicity data of female breast cancer patients who gifted tissue to the Cancer Society Tissue Bank between May 2003 and December 2007.

### Breast cancer treatment

All patients were treated following standard protocols within the Surgical and Oncology Services at Christchurch Hospital, facilitated by a weekly Breast Multidisciplinary Team Meeting. Following fine needle aspirate (FNA) or core biopsy diagnosis of breast cancer, women were staged with full blood count, biochemistry profile, and chest X-Ray. Those with clinically or pathologically determined extensive axillary lymphadenopathy, locally advanced disease, or abnormal signs, or symptoms suspicious of distant metastatic disease, underwent CT imaging of chest, abdomen and pelvis, and nuclear medicine bone scan, to rule out distant metastases. Breast surgery was wide local excision or simple mastectomy, with elective immediate reconstruction for node-negative women according to patient preference. At least level 2 axillary dissection or sentinel lymph node dissection followed by level 2 dissection for an involved sentinel node was carried out in all women. Staging was by American Joint Committee on Cancer (AJCC) TNM classification [23]. Radiation therapy was routinely given for invasive disease exceeding 40 mm diameter, 4 or more axillary nodes involved, or involved deep margins. Radiation followed chemotherapy if both modalities were given. Adjuvant chemotherapy was offered to all fit women with node-positive and poor risk node-negative tumours, using standard anthracycline/cyclophosphamide combination regimens with or without paclitaxel. Trastuzumab was added for women with HER2 positive tumours (IHC3+ or FISH amplified) following the FinHER protocol [24] according to funding by New Zealand's PHARMAC drug funding agency. Adjuvant hormonal therapy was given for all oestrogen and/or progesterone receptor positive tumours, for a minimum of 5 years. Tamoxifen was used for pre- and peri-menopausal women and aromatase inhibitors following menopause, confirmed using blood hormone levels. Follow-up was by clinical examination, 3-monthly for 2 years, then 6-monthly, with annual mammography.

### Māori-NZ European sub-cohort

A smaller sub-cohort of patients was selected for molecular studies. Breast cancer patients in the CSTB database, who had identified themselves as Māori, and who had sufficient and appropriate biological material for molecular analysis, were identified for this study. For each self-declared Māori patient, the closest match by clinicopathological data was chosen from those identifying themselves as NZ European. The characteristics of the patients that were used to match were, in order of priority: age (< 50 years or ≥ 50 years), histological type of tumour, lymph node status, oestrogen and progesterone receptor (ER and PR) status. A total of 14 Māori and 14 NZ European patients were identified, consisting of: 6 pairs under and 8 pairs over 50 years of age; 9 pairs with ductal carcinoma, 2 pairs with mixed ductal/lobular carcinoma, 1 pair with mixed ductal/tubular carcinoma and 2 mismatched pairs; 6 pairs with node negative disease, 5 pairs with 1-3 nodes involved, 1 pair with more than 3 nodes involved, and 2 mismatched pairs; 11 pairs with ER positive tumours, 2 with ER negative tumours and 1 mismatched pair; 10 pairs with PR positive tumours, 2 with PR negative tumours and 2 mismatched pairs.

### Clinicopathological data

Clinicopathological data from all 27 self-declared Māori patients and 310 self-declared European patients were analysed for the following characteristics: patient age, histological tumour type (ductal, lobular and mixed), maximum tumour size (mm), grade (1-3), metastasis to the lymph nodes (node status: N0, N1-3, N > 3), Nottingham Prognostic index (NPI = (tumour size (cm) × 0.2) + grade + node status) [25]). Grade was re-analysed by a specialist breast pathologist (GCH), blinded to ethnicity, prior histology reports and clinical data, for a random subset of patients (n = 15) from both ethnicities and was confirmed in all. Recommendations for reporting tumour prognostic factors were followed [26].

### Immunohistochemistry

Diagnostic immunohistochemistry (IHC) had been performed on samples for ER (1/200, Labvision, Hallam, Australia), PR (1/200, Novocastra, Newcastle Upon Tyne, UK) and Her2 status (Hercep test, Dako, Christchurch, NZ), as appropriate. Equivocal Her2 status was confirmed by FISH (LabPlus, Auckland, NZ).

Tissue sections for additional IHC of the sub-cohort were selected from H&E stained diagnostic slides by a specialist pathologist. Corresponding formalin-fixed, paraffin-embedded tumour tissue blocks were cut, deparaffinised and rehydrated, followed by EDTA antigen retrieval, using standard anatomical pathology procedures. Immunohistochemistry was performed on serial 3-4 μm thick sections.

IHC was performed using Cell and Tissue Staining Kits (R&D Systems, Minneapolis, MN, USA) for HIF-1α (1/25, BD Biosciences, San Jose, CA, USA), CA-IX (1/2000, Novus Biologicals, Redfurn, Australia), Glut-1 (1/200, Abcam, Cambridge, UK), cytokeratins CK5/6 (1/50, Zymed, San Francisco, USA), and CD31 (1/40, Dako, Christchurch, NZ) following manufacturers' protocols. Staining was followed by automated counterstaining with hematoxylin (Leica Autostainer XL). Two slides were stained per antibody and each was read twice by a pathologist on two separate occasions, according to statistical advice.

### Quantification of IHC

Receptor status data was available through the CSTB database, and presented as follows: QuickScore (QS) as a score out of 8 measuring intensity and area [27], and Histoscore (H-score) as a score out of 300: intensity of staining on a scale of 0-3, and a percentage (0-100) area stained [28]. For statistical analysis Her2 status was separated into positive or negative, and ER/PR status was separated into negative (QS = 0, H-score = 0), weak/moderate positive (QS = 1-6, H-score = 1-150) and strong positive (QS = 7-8, H = 151-300).

Immunostaining was evaluated by an independent pathologist who was blinded to patient clinico-pathological data, including ethnicity. IHC staining for all hypoxia markers was quantified using H-score. For HIF-1α, only staining of the nucleus of tumour cells was considered. Cytoplasmic and membrane staining of tumour cells was considered for Glut-1 and CA-IX. Cytokeratin CK5/6 staining was scored as positive or negative. Microvessel density (MVD) according to CD31 positive cells was quantified as described before [29]. Briefly, slides were scanned at low power (x40-100) for three areas of highest vascularity, then were examined at high power (x250-400) and placed into high vs. medium/low categories.

### Immunoassay of serum samples

VEGF-A and IGF-1 levels in serum samples of the sub-cohort of breast cancer patients were measured using Quantikine Human Immunoassay kits (R&D Systems), following manufacturer's recommendations. Serum samples were assayed in duplicate on two separate occasions with associated standards as controls. Of the corresponding tumour samples, 22/28 breast cancer serum samples in the sub-cohort were available for analysis.

### Statistics

For the entire cohort, ER and PR status (negative, weak to moderate and strong positive) in the two ethnic groups was compared using chi-square tests for contingency tables. Ordinal categories were compared using proportional odds logistic regression. If the proportional odds assumption was violated, a Cochran-Armitage test for trend was used. Continuous variables were compared using t-tests. Tumour size had a skewed distribution and so both square root and log transformations were explored. Patient survival was calculated from the date of primary surgery to the date of death or the last follow-up according to the Kaplan-Meier method. Disease-free survival was calculated similarly with date of detection of metastases as the endpoint. Differences in survival distributions were evaluated by a log-rank test. Values of p < 0.05 were considered significant.

For the matched subcohort, comparisons between the two ethnic groups used paired t-tests for continuous variables, McNemar's test for the significance of changes for binary variables and Bowker's test of symmetry for ordered categories. Within the matched-cohort it was necessary to take account of the matching when examining relationships between clinico-pathological variables and serum- and tumour-associated factors, and correlations among the serum- and tumour-associated factors. This was done by treating each matched pair as a cluster and carrying out analyses in SUDAAN using jackknifing to obtain appropriate standard errors and p values.

All other analyses were carried out in SAS 9.1.

## Results

### Breast cancer patient cohort

During the period of 2003-2007 the CSTB had accumulated samples gifted from 357 breast cancer patients with associated ethnicity data (Table 1). Data showed that 7.6% of breast cancer donors identified themselves as Māori and 86.8% as European (NZ European and other European). Mean age at diagnosis of Māori patients was 3.4 years younger than that of European patients, but this was not significant (p = 0.63) (Table 2).

**Table 2:** Clinicopathological characteristics of breast cancer patients.

Measure	European (n = 310)	Māori (n = 27)	Ethnic difference (95% CI)	Statistical test^1^	Test value	df	p
**Age** (years)	mean 61.1 (sd 14.4)	mean 57.7 (sd 13.3)	3.4 years (-2.2, 9.1)	t test	1.19	335	0.63
**Ductal**	74.2% (230)	66. 7% (18)	% ductal	ductal vs rest χ^2^	0.72	1	0.39
**Lobular**	14.8% (46)	22.2% (6)	7.5%				
**Other**	11.0% (34)	11.1% (3)	(-10.9, 26.0)				
**Unknown**	0	0					
**Tumour** **size **(mm)	mean 21.5 (sd 13.2)	mean 23.6 (sd 14.4)	-2.1 mm (-7.3, 3.2)	t test	-0.78	332	0.43
**unknown**	3	0					
**Grade: 1**	13.0% (40)	18.5% (5)	% Grade 3 vs 1 or 2	Wald χ^2^	4.77	1	0.027*
**2**	43.7% (134)	63.0% (17)	24.8%				
**3**	43.3% (133)	18.5% (5)	(9.1, 40.5)				
**unknown**	3	0					
**Node: N0**	52.8% (160)	40.0% (10)	Any +ve	Wald χ^2^	1.73	1	0.19
**N1-3**	31.0% (94)	36.0% (9)	-12.8%				
**N > 3**	16.2% (49)	24.0% (6)	(-32.8, 7.2)				
**unknown**	7	2					
**NPI**	mean 4.4 (sd 1.3)	mean 4.3 (sd 1.4)	0.05 (-0.5, 0.6)	t test	0.18	326	0.85
**unknown**	7	2					
**ER: -ve**	19.7% (60)	11.5% (3)	% Strong	Wald χ^2^	2.78	1	0.095
**weak +ve**	10.8% (33)	0% (0)	-19.0%				
**strong +ve**	69.5% (212)	88.5% (23)	(-32.3, -5.6)				
**unknown**	5	1					
**PR: -ve**	31.0% (94)	23.1% (6)	% Strong	Wald χ^2^	1.65	1	0.20
**weak +ve**	36.0% (109)	30.8% (8)	-13.2%				
**strong +ve**	33.0% (100)	46.1% (12)	(-33.0, 6.7)				
**unknown**	7	1					
**Her2: +ve**	15.0% (25)	12.5% (2)	% +ve	χ^2^	0.07	1	0.79
**-ve**	85.0% (142)	87.5% (14)	2.5				
**unknown**	143	11	(-14.6, 19.6)				

Clinicopathological characteristics of all Māori and European female breast cancer patients who gifted tissue to the Cancer Society Tissue Bank between May 2003 and December 2007 (sd = standard deviation, df = degrees of freedom). *significant at p < 0.05.

^1 ^t test for two independent samples (pooled variance as no indication from folded F of unequal variances), χ^2 ^from chi-square test for contingency tables, Wald χ^2 ^from ordinal logistic regression for which the proportional odds assumption was not violated.

A higher proportion of Māori patients presented with grade 2 than grade 3 tumours, whereas a similar proportion of European patients had grade 2 and 3 tumours. Māori patients had odds ratio of 0.4 of European patients of a higher grade tumour (p = 0.027) (Table 2). A marginal difference in ER status between Māori and European patients was apparent (p = 0.095), with a tendency for Māori patients to have a positive ER status. Similar proportions of tumours were negative, weak or positive for PR, with no ethnic differences. No differences in node status, histological tumour type, maximum tumour size or NPI were apparent between the two ethnicities. However, European women had tumours on average 2.1 mm smaller than Māori women, and a lower percentage with any positive nodes (47.2% vs. 60.0%) (Table 2).

Her2 status had not been determined for 46% of all tumours, in accordance with local protocols at the time. Her2 status was known for 95% of patients under 50 years of age, and for 41% of patients over 50 years of age. Of those patients with known Her2 status (n = 186) the intrinsic subtypes were as follows: 1 Māori and 34 European were triple negative (Her2-, ER/PR-), 13 Māori and 111 European were luminal A (Her2-, ER and/or PR+), 1 Māori and 13 European were luminal B (Her2+, ER and/or PR+), and 1 Māori and 12 European were Her2 (Her2+, ER/PR-).

**Figure 1:**
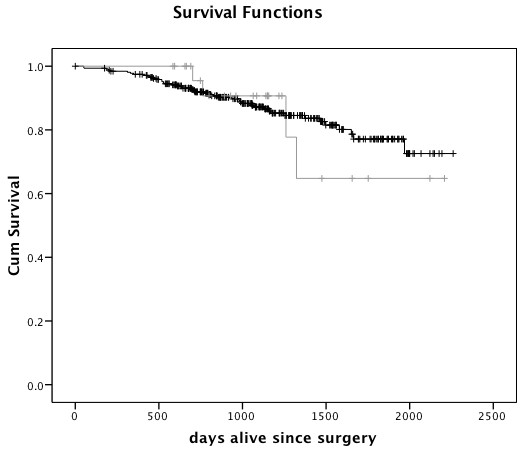
**Breast cancer survival in Māori and European women**. Survival from surgery to death from any cause by Kaplan-Meier survival analysis of Māori (n = 27, grey) and European (n = 310, black) women with breast cancer.

**Figure 2:**
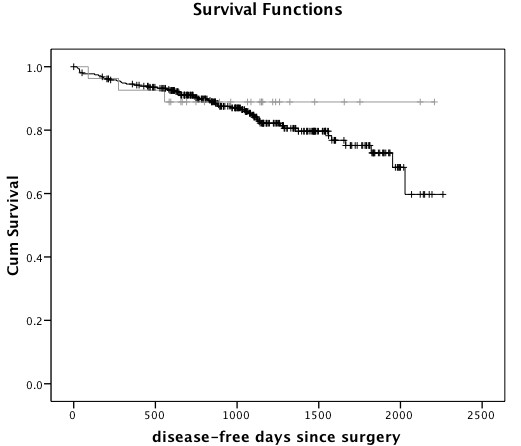
**Disease-free survival of Māori and European women with breast cancer**. Kaplan-Meier survival analysis of Māori (n = 27, grey) and European (n = 310, black) women with breast cancer.

Median follow-up time for Māori and European women was similar (1085 and 1061 days, respectively). Four Māori patients and 42 European patients had died during follow-up, and 3 Māori and 50 European women had a recorded recurrence or metastasis during follow up (up to 14^th ^September 2009). Kaplan-Meier survival analysis showed no significant difference in overall survival (log rank, p = 0.86) (Figure 1) or disease-free survival (log rank, p = 0.50) (Figure 2) between Māori and European breast cancer patients. Median survival was not reached, i.e. more than 50% of the cohort were alive and/or disease-free at the end of follow up. No further recurrences were observed in the Māori group after 556 days following surgery, while metastases continued to be recorded in the European group for up to 2028 days. Death in Māori women was recorded up to 1323 days after surgery, and for up to 1969 days in European women. The low number of Māori patients who had recorded recurrence/metastasis or death (total n = 5) limited significant conclusions.

### Molecular data in sub-cohort of breast cancer patients

Due to careful matching, there were no significant ethnic differences by clinicopathological criteria in the sub-cohort (n = 28). Due to low patient numbers in this sub-cohort, ethnic differences could not be clarified, and no statistically significant differences were observed between the two ethnic groups for IHC staining for either intrinsic hypoxia markers (HIF-1α p = 0.98, GLUT-1 p = 0.69, CA-IX p = 0.61), or microvascular density (p = 0.65), or cytokeratins (CK5/6 p = 0.65) in tumours (Table 3). Similarly, there were no significant differences in circulating factors in serum from Māori and NZ European cancer patients for either VEGF-A (p = 0.10) or IGF-1 (p = 0.27).

**Table 3:** Molecular data of Māori and NZ European sub-cohort of breast cancer patients.

	Māori **no**.	Māori	NZ European **no**.	NZ European	Mean difference (95% CI)	t^a^	df^b^	p
**HIF-1α** H-score ^c^	14	75.5 (± 55.5)	14	75.2 (± 58.9)	0.36 (-34.4, 35.1)	0.02	13	0.98
**GLUT-1** H-score ^c^	14	112.9 (± 45.2)	14	120.4 (± 46.4)	-7.5 (-47.6, 32.6)	-0.40	13	0.69
**CA-IX** H-score^c^	14	56.6 (± 32.6)	14	64.0 (± 59.1)	-7.4 (-37.7, 22.9)	-0.53	13	0.61
**MVD** high/low^d^	14	5/14 (35.7%)	14	6/14 (42.9%)	7.2%	0.2	1	0.65
**CK5/6** pos/neg ^d^	14	2/14 (14.3%)	14	3/14 (21.4%)	7.1%	0.2	1	0.65
**VEGF-A** pg/ml ^c^	11	299.9 (± 162.7)	11	359.6 (± 174.8)	-59.8 (-134, 14.5)	-1.79	10	0.10
**IGF-1** ng/ml ^c^	11	114.5 (± 31.4)	11	100.6 (± 24.7)	14.0 (-12.4, 40.4)	1.18	10	0.27

^a ^t from a t-test for paired samples or S from McNemar's test for significance of changes

^b ^degrees of freedom

^c ^mean (± sd)

^d ^frequency (%) high/positive

HIF-1α was expressed in 93% of the breast tumours, and all samples showed some degree of GLUT-1 and CA-IX staining. In tumour and vascular endothelial cells, both nuclear and cytoplasmic staining of HIF-1α was observed, as well as patchy, diffuse and increased staining adjacent to necrosis, but only nuclear staining of tumour cells was quantified. GLUT-1 and CA-IX staining showed patchy as well as increased expression adjacent to necrosis. CK5/6 staining was very low or absent in most tumour samples. High microvascular density was detected in 40% of samples.

Comparisons of molecular factors and clinicopathological details showed several significant relationships (Table 4). High nodal status correlated with reduced CA-IX (p = 0.0068). Grade showed a positive association with HIF-1α (p = 0.0064), GLUT-1 (p = 0.013), MVD (0.0009) and CK5/6 (p = 0.0069). Negative ER status was associated with increased GLUT-1 (p = 0.046) and high MVD (p = 0.038). PR status showed similar associations with GLUT-1 (p = 0.024), CA-IX (p = 0.018) and MVD (p = 0.013).

**Table 4:** Molecular data of matched Māori and European sub-cohort of breast cancer patients arranged by predictive clinicopathological factors.

Measure (n=)	HIF-1α H-score Mean (± sd)	GLUT-1 H-score Mean (± sd)	CA-IX H-score Mean (± sd)	MVD frequency high (%)	CK 5/6 frequency positive (%)	VEGF-A pg/ml Mean (± sd)	IGF-1 ng/ml Mean (± sd)
**Nodal status^1^**	0.073	0.22	**0.0068**	0.65	0.10	0.40	0.77
**N0 **(12)	44.38 (± 27.08)	107.08 (± 43.47)	63.65 (± 24.79)	1/12 (8.33%)	1/12 (8.33%)	323.39 (± 167.41)	103.2 (± 22.27)
**N1 **(12)	100.21 (± 67.46)	131.88 (± 46.19)	64.17 (± 67.78)	8/12 (66.7%)	4/12 (33.33%)	370.00 (± 189.65)	109.93 (± 35.57)
**N2 **(4)	93.75 (± 47.72)	99.38 (± 42.93)	38.75 (± 7.77)	2/4 (50%)	0/4 (0.0%)	230.13 (± 50.22)	114.67 (± 32.40)
**Grade^1^**	**0.0064**	**0.013**	0.075	**0.0009**	**0.0069**	0.15	0.29
**1 **(5)	41.5 (± 19.57)	108.50 (± 31.60)	72.5 (± 19.04)	0/5 (0.0%)	0/5 (0.0%)	348.75 (± 126.36)	102.03 (± 21.77)
**2 **(13)	50.96 (± 36.29)	94.23 (± 37.63)	42.31 (± 16.09)	2/13 (15.38%)	0/13 (0.0%)	401.92 (± 209.77)	119.19 (± 34.50)
**3 **(10)	124.0 (± 58.47)	149.75 (± 42.22)	77.63 (± 72.38)	9/10 (90.0%)	5/10 (50%)	236.67 (± 89.36)	97.92 (± 22.48)
**ER status^1^**	0.18	**0.046**	0.052	**0.038**	0.18	0.14	0.075
**Negative **(5)	131.50 (± 70.83)	142.0 (± 53.19)	120.50 (± 73.56)	5/5 (100%)	2/5 (40%)	245.98 (± 109.29)	87.49 (± 17.57)
**Positive **(23)	63.15 (± 45.72)	111.09 (± 42.43)	47.23 (± 26.94)	6/23 (26.09%)	3/23 (13.04%)	354.38 (± 176.18)	113.46 (± 28.75)
**PR status^1^**	0.15	**0.024**	**0.018**	**0.013**	0.059	0.069	0.074
**Negative **(6)	122.08 (± 67.42)	151.25 (± 52.70)	118.13 (± 66.05)	6/6 (100%)	3/6 (50%)	237.44 (± 99.96)	90.77 (± 17.66)
**Positive **(22)	62.61 (± 46.72)	107.16 (± 38.92)	44.54 (± 24.22)	5/22 (22.73%)	2/22 (9.09%)	364.36 (± 176.92)	113.85 (± 29.64)

^1^Overall comparisons of outcomes for categorical predictors are shown; significant comparisons are shown in bold (p < 0.05). Analysis took account of the paired design by treating each pair as a cluster and using jackknifing of clusters, in SUDAAN.

A comparison among molecular factors showed strong positive correlations between HIF-1α and Glut-1 (p = 0.001), MVD (p = 0.01), and CK5/6 (p = 0.002). CK5/6 correlated positively with Glut-1 (p < 0.0001) and MVD (p = 0.001) (Table 5).

**Table 5:** Associations of tumour and circulating factors in breast cancer patients.

	**Correlations**^**1**^	GLUT-1	CA-IX	MVD	CK5/6	VEGF-A	IGF-1
**HIF-1α**	r (p)	**0.66** **(0.001)**	0.45 (0.27)	**0.57** **(0.01)**	**0.59** **(0.002)**	-0.16 (0.45)	-0.15 (0.63)
**GLUT-1**	r (p)		0.40 (0.11)	**0.38** **(0.03)**	**0.73** **(< 0.0001)**	0.06 (0.82)	-0.00 (0.98)
**CA-IX**	r (p)			0.25 (0.36)	0.34 (0.27)	0.01 (0.95)	-0.35 (0.23)
**MVD**	r (p)				**0.58** **(0.001)**	0.41 (0.10)	0.29 (0.18)
**CK5/6**	r (p)					0.27 (0.12)	0.17 (0.12)
**VEGF-A**	r (p)						-0.03 (0.96)

Associations of tumour-associated (HIF-1α, GLUT-1, CA-IX, MVD, CK5/6) and circulating (VEGF-A, IGF-1) factors in sub-cohort of breast cancer patients (n = 28). Pearson product moment correlations, 2-tailed, reported as r, with p in brackets, significant correlations are shown in bold p < 0.05 (VEGF-A n = 22, and IGF-1 n = 21).

^1^Analysis took account of the paired design by treating each pair as a cluster and using jackknifing of clusters, in SUDAAN.

Circulating molecular factors in serum did not show significant associations with any clinicopathological or tumour associated factors. However, VEGF-A concentrations tended to be higher in patients with PR positive tumours (p = 0.069), and IGF-1 higher in ER/PR positive tumours (p = 0.075/0.074) (Table 4).

Median follow-up time for Māori and NZ European women in the sub-cohort was similar (1227 and 1281 days, respectively). Of the 14 Māori patients, 3 died within the follow-up period, whereas none of the NZ European patients died. Two of the NZ European women had a recorded metastasis in the follow-up period, but none of the Māori women did.

## Discussion

Analysis of clinical, pathological and molecular data of the CSTB cohort of breast cancer patients found no evidence of worse prognostic indicators in self-declared Māori compared to self-declared European women. Furthermore, our study demonstrated that Māori breast cancer patients presented with significantly lower grade tumours than European breast cancer patients, which is in contrast to other published reports from elsewhere in New Zealand [11,12,18,30,31].

Specifically, a recent report by the Auckland Breast Cancer Study Group showed that NZ Māori participants (n = 133) had higher grade tumours than European participants (n = 1220) [18]. Weston et al. also demonstrated that NZ Māori participants had larger tumours with more involved lymph nodes than European participants [18], which is supported by an analysis of prognostic factors of 21,586 breast cancer cases in the New Zealand Cancer Registry, showing that tumours in Māori women were more likely to be larger, less well differentiated (grade), Her2 positive, and ER and PR negative than tumours in non- Māori/non-Pacific women [31]. Our data showed no significant difference in tumour size (upper 95% CI showed a difference of 7.3 mm) which could have indicated a difference in delay in presentation. Node status, which could have indicated a difference in extent of disease spread, was not significantly different either, although 95% CI were well within the estimates from national studies [18,31]. Receptor status was somewhat more favourable for Māori women and not compatible with a major disadvantage for them. Survival did not differ between the two ethnicities during the limited follow-up period, which is in accord with our clinicopathological and molecular data.

The majority of tumours (with known Her2 status) from both ethnicities were intrinsic subtype luminal A. Different prevalence of intrinsic subtypes of breast cancer in different ethnic groups in the US had been reported [32]. Compared to Caucasians, African Americans showed a higher, whereas Japanese showed a lower prevalence of basal-like breast tumour subtype (triple negative and cytokeratin 5/6+ and/or HER1+), with associated poorer prognosis for African American women and better prognosis for Japanese women [32,33].

Clearly, the main limitation of our study is the small number of patient samples (n = 337). The constraint is due to availability of samples from breast cancer patients who identified themselves as Māori (n = 27). Molecular research is of concern for indigenous people, and needs to be carried out with cultural sensitivity. In New Zealand, Māori traditionally consider that human tissue and the information derived from it, is collectively owned by the whānau (family), hapū (sub-tribe) and iwi (tribe). Hence, tissue donation may require iwi and whānau approval. The accrual of Māori samples by the CSTB, which holds the largest collection of tumour samples for research in New Zealand, is considered an achievement [9,10]. The samples analysed in this study represent all available samples from Māori breast cancer patients since accurate ethnicity data collection was introduced in 2003 up to the end of 2007.

This study was performed specifically with breast cancer patients treated through Christchurch Hospital in Canterbury, South Island NZ. Reports into ethnic inequalities in cancer mortality do not report data by region [11] and studies into regional inequalities in cancer mortality do not report ethnicity data [34]. The Ministry of Health's Atlas of Cancer Mortality (1994-2000) has 'insufficient' ethnicity data ('less than 16 cancer deaths over 7 year time period') at the level of District Health Board for most of the South Island for most cancer types, and no regional data on breast cancer mortality by ethnicity is available for Canterbury [35]. Hence, as 87% of Māori live in the North Island [36], all national data on ethnic inequalities in cancer incidence, mortality and survival are necessarily shaped by data from the North Island. This may partly explain why our data differs from previous publications, but also urges further research into potential regional variability.

It is of note that most published epidemiological data on breast cancer have utilised the New Zealand Cancer Registry data set. A recent paper has quantified the discrepancy between ethnicity data of the registry and census statistics between 1981 and 2004 [37]. Māori were initially undercounted by as much as 31% in the registry compared to self-reported data, but even the 2001 data showed undercounting of Māori by 15%. Hence most recent studies incorporate adjustment factors to take account of undercounting (e.g. [15]).

The selection of breast cancer patients may account for differences in the studies. All patients attending the Christchurch Hospital pre-admission clinic, prior to cancer surgery, are considered potential donors, and are given the opportunity to donate part of their tumour to research [9]. However, the CSTB currently only consents breast cancer patients who attend Christchurch Hospital, and not those who are treated through the private sector. In addition, only patients who have operable cancers of sufficient size are consented, and only tumour samples excess to diagnosis can be banked, thus possibly selecting for larger tumours. On the other hand, the percentage of patients who identified themselves as Māori in this study (7.4%) compared well with the New Zealand census data for the Canterbury region, which showed that 7.2% of people identified themselves as Māori [38], indicating that this cohort is representative of the region.

This is the first study to analyse hypoxia-related prognostic factors in a sub-cohort of Māori breast cancer patients. Due to low numbers it could not clarify ethnic differences between the groups. However, it demonstrated significant associations between clinicopathological data and several molecular factors in tumours and serum across both ethnicities, confirming their strong predictive value even in this small cohort. A significant association of CK5/6 with microvessel density and hypoxia (HIF-1α and GLUT-1) was detected, a first report to our knowledge. Presence of CK5/6 is a hallmark of basal-like carcinoma, which is an aggressive form of breast cancer with, reportedly, the worst prognosis of all molecular breast cancer subtypes [39,40]. However, the prognostic significance of CK5/6 expression is controversial, as some studies have shown that poor prognosis was determined by ER absence and not by CK5/6 expression [41]. In a group of 30 hereditary breast cancer patients, an association between HIF-1α and CK5/6 was indicated, but did not reach significance [42]. Our study did not confirm the reported association between CK5/6 and CA-IX [43].

Correlations detected in this study of hypoxia markers with nodal status and grade confirm previous studies in breast cancer [e.g. [44,45]]. Our study supported an inverse relationship between tumour hypoxia and hormone receptor status, although the relationship between hypoxia markers and ER status remains controversial [46-49].

## Conclusions

This study could find no evidence of poorer prognostic indicators in Māori compared to European women with breast cancer in the Canterbury region, suggesting regional differences within New Zealand. As regional ethnicity data on cancer is extremely sparse, further studies are warranted. Future studies are supported by the launch in August 2009 of the Christchurch Breast Cancer Patient Register. Our data may also assist a recent memorandum of understanding which was signed between Manawhenua ki Waitaha, a representative collective of the seven Ngāi Tahu Rūnanga (Māori tribal organisation/authority), and the Canterbury District Health Board to assist in improving the health outcomes for the Canterbury Māori population [50].

## Competing interests

The authors declare that they have no competing interests.

## Authors' contributions

GUD conceived and designed the overall study, acquired some of the clinical data, made substantial contribution to the analysis of the data, drafted and revised the initial manuscript, and gave ultimate approval of the final manuscript. MK contributed to the design, acquisition and analysis of data of the sub-cohort study, revised and gave final approval of the manuscript. EV contributed to the acquisition and analysis of data of the clinical and sub-cohort study, revised and gave final approval of the manuscript. HRM contributed to the acquisition of data of the study, critically revised and gave final approval of the manuscript. VCLD contributed to the acquisition of survival data of the study, revised and gave final approval of the manuscript. GCH acted as expert pathologist in gathering and interpreting pathology data, and revised and gave final approval of the manuscript. MC contributed to the acquisition of data of the clinical and sub-cohort study, revised and gave final approval of the manuscript. CF acted as biostatistician in the design of the subcohort study and statistical interpretation of this data, and revised and gave final approval of the manuscript. MJC contributed to the design and interpretation of data of the study, revised and gave final approval of the manuscript. JEW acted as expert biostatistician in the overall design and statistical interpretation of data, and critically revised intellectual content, and gave final approval of the manuscript. BAR contributed to the design and interpretation of all clinical data of the study, critically revised intellectual and clinical content, and gave final approval of the manuscript.

## Pre-publication history

The pre-publication history for this paper can be accessed here:

http://www.biomedcentral.com/1471-2407/10/543/prepub
